# Deep Convolutional and LSTM Recurrent Neural Networks for Multimodal Wearable Activity Recognition

**DOI:** 10.3390/s16010115

**Published:** 2016-01-18

**Authors:** Francisco Javier Ordóñez, Daniel Roggen

**Affiliations:** Wearable Technologies, Sensor Technology Research Centre, University of Sussex, Brighton BN1 9RH, UK; daniel.roggen@ieee.org

**Keywords:** human activity recognition, wearable sensors, deep learning, machine learning, sensor fusion, LSTM, neural network

## Abstract

Human activity recognition (HAR) tasks have traditionally been solved using engineered features obtained by heuristic processes. Current research suggests that deep convolutional neural networks are suited to automate feature extraction from raw sensor inputs. However, human activities are made of complex sequences of motor movements, and capturing this temporal dynamics is fundamental for successful HAR. Based on the recent success of recurrent neural networks for time series domains, we propose a generic deep framework for activity recognition based on convolutional and LSTM recurrent units, which: (i) is suitable for multimodal wearable sensors; (ii) can perform sensor fusion naturally; (iii) does not require expert knowledge in designing features; and (iv) explicitly models the temporal dynamics of feature activations. We evaluate our framework on two datasets, one of which has been used in a public activity recognition challenge. Our results show that our framework outperforms competing deep non-recurrent networks on the challenge dataset by 4% on average; outperforming some of the previous reported results by up to 9%. Our results show that the framework can be applied to homogeneous sensor modalities, but can also fuse multimodal sensors to improve performance. We characterise key architectural hyperparameters’ influence on performance to provide insights about their optimisation.

## 1. Introduction

Recognizing human activities (e.g., from simple hand gestures to complex activities, such as “cooking a meal”) and the context in which they occur from sensor data is at the core of smart assistive technologies, such as in smart homes [1], in rehabilitation [2], in health support [3,4], in skill assessment [5] or in industrial settings [6]. Some simple activity-aware systems are now commercial in the form of fitness trackers or fall detection devices. However, many scenarios of high societal value are still elusive, such as providing “memory prosthesis” to people with dementia, inserting subtle cues in everyday life in the right context to support voluntary behaviour change (e.g., to fight obesity), or enabling natural human-robot interaction in everyday settings. These scenarios require a minute understanding of the activities of the person at home and out and about.

This work is motivated by two requirements of activity recognition: enhancing recognition accuracy and decreasing reliance on engineered features to address increasingly complex recognition problems. Human activity recognition is challenging due to the large variability in motor movements employed for a given action. For example, the OPPORTUNITYchallenge that was run in 2011 aiming at recognising activities in a home environment showed that contenders did not reach an accuracy higher than 88% on the recognition of only 17 sporadic gestures [7]. Thus, addressing scenarios, such as activity diarisation, will require further improving recognition performance for an even wider set of activities.

Human activity recognition (HAR) is based on the assumption that specific body movements translate into characteristic sensor signal patterns, which can be sensed and classified using machine learning techniques. In this article, we are interested in wearable (on-body) sensing, as this allows activity and context recognition regardless of the location of the user.

Wearable activity recognition relies on combinations of sensors, such as accelerometers, gyroscopes or magnetic field sensors [8]. Patterns corresponding to activities are then detected within the streaming sensor data using either feature extraction on sliding windows followed by classification, template matching approaches [9] or hidden Markov modelling [10]. Sliding window approaches are commonly used for static and periodic activities, while sporadic activities lend themselves to template matching approaches or hidden Markov modelling [8,11].

Most recognition systems select features from a pool of “engineered” features [12]. Identifying relevant features is time consuming and also leads to a difficulty in “scaling up” activity recognition to complex high level behaviours (e.g., hour-long, day-long or more), as engineered features do not relate to “units of behaviour”, but are rather the result of convenient mathematical operations. For instance, statistical and frequency features do not relate to semantically meaningful aspects of human motion, such as “hand grasp”.

Deep learning refers broadly to neural networks that exploit many layers of non-linear information processing for feature extraction and classification, organised hierarchically, with each layer processing the outputs of the previous layer. Deep learning techniques have outperformed many conventional methods in computer vision [13] and audio classification [14].

Convolutional neural networks (CNNs) [15] are a type of DNN (deep neural network) with the ability to act as feature extractors, stacking several convolutional operators to create a hierarchy of progressively more abstract features. Such models are able to learn multiple layers of feature hierarchies automatically (also called “representation learning”). Long-short-term memory recurrent (LSTMs) neural networks are recurrent networks that include a memory to model temporal dependencies in time series problems. The combination of CNNs and LSTMs in a unified framework has already offered state-of-the-art results in the speech recognition domain, where modelling temporal information is required [16]. This kind of architecture is able to capture time dependencies on features extracted by convolutional operations.

Deep learning techniques are promising to address the requirements of wearable activity recognition. First, performance may chiefly be improved over existing recognition techniques. Second, deep learning approaches may have the potential to uncover features that are tied to the dynamics of human motion production, from simple motion encoding in lower layers to more complex motion dynamics in upper layers. This may be useful to scaling up activity recognition to more complex activities.

The contributions of this paper are the following:

- We present DeepConvLSTM: a deep learning framework composed of convolutional and LSTM recurrent layers, that is capable of automatically learning feature representations and modelling the temporal dependencies between their activation.
- We demonstrate that this framework is suitable for activity recognition from wearable sensor data by using it on two families of human activity recognition problems, that of static/periodic activities (modes of locomotion and postures) and that of sporadic activities (gestures).
- We show that the framework can be applied seamlessly to different sensor modalities individually and that it can also fuse them to improve performance. We demonstrate this on accelerometers, gyroscopes and combinations thereof.
- We show that the system works directly on the raw sensor data with minimal pre-processing, which makes it particularly general and minimises engineering bias.
- We compare the performance of our approach to that reported by contestants participating to a recognised activity recognition challenge (OPPORTUNITY) and to another open dataset (Skoda).
- We show that the proposed architecture outperforms published results obtained on the OPPORTUNITY challenge, including a deep CNN, which had already offered state-of-the-art results in previous studies [17].
- We discuss the results, including the characterisation of key parameters’ influence on performance, and outline venues for future research towards taking additional advantages of the characteristics of the deep architecture.

## 2. State of the Art

Neural networks are powerful for pattern classification and are at the base of deep learning techniques. We introduce the fundamentals of shallow recurrent networks in Section 2.1, in particular those built on LSTM units, which are well suited to model temporal dynamics. In Section 2.2, we review the use of deep networks for feature learning, in particular convolutional networks. In Section 2.3, we review the use of neural networks in deep architectures and their applications to domains related to human activity recognition.

### 2.1. From Feedforward to Recurrent Networks

A feedforward neural network, or multi-layer perceptron (MLP), is a computational model that processes information through a series of interconnected computational nodes. These computational nodes are grouped into layers and are associated with one another using weighted connections. The nodes of the layers are called units (or neurons) and transform the data by means of non-linear operations to create a decision boundary for the input by projecting it into a space where it becomes linearly separable.

**Figure 1:**
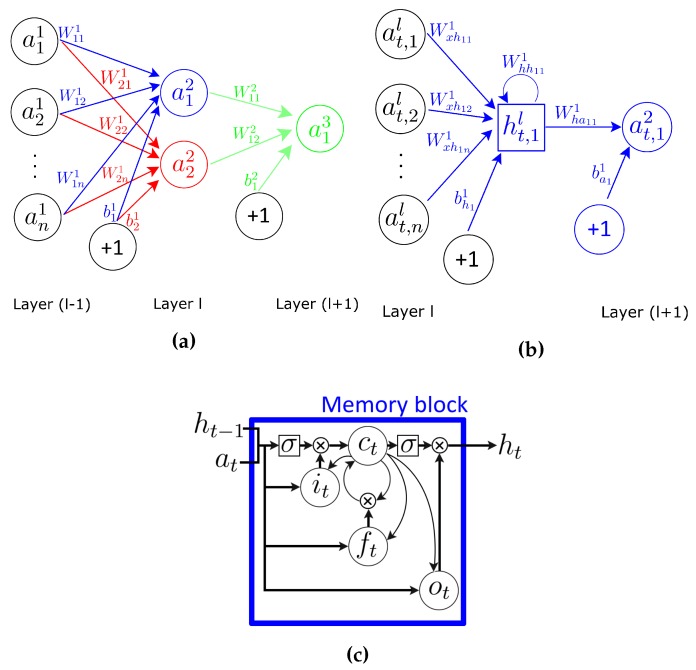
Different types of units in neural networks. (**a**) MLP with three dense layers; (**b**) recurrent neural network (RNN) with two dense layers. The activation and hidden value of the unit in layer (l+1) are computed in the same time step *t*; (**c**) The recurrent LSTM cell is an extension of RNNs, where the internal memory can be updated, erased or read out.

MLPs have been successfully applied to classification problems by training them in a supervised manner. Every neuron can be viewed as a computational unit that behaves as a logistic regression classifier (see Figure 1a). Formally, units are defined in terms of the following function:

(1) $$a^{\left(l+1\right)}=\sigma \left(W^{l}a^{l}+b^{l}\right)$$

where $a^{l}$ denotes the level of response (or activation value) for the units in layer *l* ($a_{i}^{l}$ is the activation for the unit *i* in layer *l*), *W* is a weight matrix, where $W_{ij}^{l}$ represents the parameter (or weight) associated with the connection between unit *j* in layer *l*, and unit *i* in layer l+1, $b^{l}$ is the bias associated with units in layer *l* and *σ* is the activation function (or non-linearity). For l=1, we use $a^{\left(1\right)}=x$, denoting the input data of the network (the sensor signal in our problem). The output of an MLP architecture is defined by the activations of the units in the deepest layer. MLPs use a fully-connected (or dense) topology, in which each unit in layer (l+1) is connected with every unit in layer *l*.

A limitation of the MLP architecture is that it assumes that all inputs and outputs are independent of each other. In order for an MLP to model a time series (such as a sensor signal), it is necessary to include some temporal information in the input data. Recurrent neural networks (RNNs) are neural networks specifically designed to tackle this problem, making use of a recurrent connection in every unit. The activation of a neuron is fed back to itself with a weight and a unit time delay, which provides it with a memory (hidden value) of past activations, which allows it to learn the temporal dynamics of sequential data. A representation of a single recurrent unit is shown in Figure 1b.

Given a temporal input sequence $a^{l}=\left(a_{1}^{l},\cdots ,a_{T}^{l}\right)$ of length *T* (being $a_{t,i}^{l}$ the activation of the unit *i* in hidden layer *l* at time *t*), an RNN maps it to a sequence of hidden values $h^{l}=\left(h_{1}^{l},\cdots ,h_{T}^{l}\right)$ and outputs a sequence of activations $a^{\left(l+1\right)}=\left(a_{1}^{\left(l+1\right)},\cdots ,a_{T}^{\left(l+1\right)}\right)$ by iterating the following recursive equation:

(2) $$h_{t}^{l}=\sigma \left(W_{xh}^{l}a_{t}^{l}+h_{t-1}^{l}W_{hh}^{l}+b_{h}^{l}\right)$$

where *σ* is the non-linear activation function, $b_{h}^{l}$ is the hidden bias vector and *W* terms denote weight matrices, $W_{xh}^{l}$ being the input-hidden weight matrix and $W_{hh}^{l}$ the hidden-hidden weight matrix. The activation for these recurrent units is defined by:

(3) $$a_{t}^{\left(l+1\right)}=h_{t}^{l}W_{ha}^{l}+b_{a}^{l}$$

where $W_{ha}^{l}$ denotes the hidden-activation weight matrix and the $b_{a}^{l}$ terms denote the activation bias vector. Notice that the weight matrix $W^{l}$ defined for the MLP is equivalent to the $W_{xh}^{l}$ matrix in Equation (2).

These types of networks have Turing capabilities [18] and, thus, are in principle suited for learning sequences. However, their memory mechanism makes learning challenging when dealing with real-world sequence processing [19].

LSTMs extend RNN with memory cells, instead of recurrent units, to store and output information, easing the learning of temporal relationships on long time scales. LSTMs make use of the concept of gating: a mechanism based on component-wise multiplication of the input, which defines the behaviour of each individual memory cell. The LSTM updates its cell state, according to the activation of the gates. The input provided to an LSTM is fed into different gates that control which operation is performed on the cell memory: write (input gate), read (output gate) or reset (forget gate). The activation of the LSTM units is calculated as in the RNNs (see Equation (2)). The computation of the hidden value $h_{t}$ of an LSTM cell is updated at every time step *t*. The vectorial representation (vectors denoting all units in a layer) of the update of an LSTM layer is as follows:

(4) $$i_{t}=\sigma_{i}\left(W_{ai}a_{t}+W_{hi}h_{t-1}+W_{ci}c_{t-1}+b_{i}\right)$$

(5) $$f_{t}=\sigma_{f}\left(W_{af}a_{t}+W_{hf}h_{t-1}+W_{cf}c_{t-1}+b_{f}\right)$$

(6) $$c_{t}=f_{t}c_{t-1}+i_{t}\sigma_{c}\left(W_{ac}a_{t}+W_{hc}h_{t-1}+b_{c}\right)$$

(7) $$o_{t}=\sigma_{o}\left(W_{ao}a_{t}+W_{ho}h_{t-1}+W_{co}c_{t}+b_{o}\right)$$

(8) $$h_{t}=o_{t}\sigma_{h}\left(c_{t}\right)$$

where i, f, o and c are respectively the input gate, forget gate, output gate and cell activation vectors, all of which are the same size as vector h defining the hidden value. Terms *σ* represent non-linear functions. The term $a_{t}$ is the input to the memory cell layer at time *t*. $W_{ai},W_{hi},W_{ci},W_{af},W_{hf},W_{cf},W_{ac},W_{hc},W_{ao},W_{ho}$ and $W_{co}$ are weight matrices, with subscripts representing from-to relationships ($W_{ai}$ being the input-input gate matrix, $W_{hi}$ the hidden-input gate matrix, and so on). $b_{i},b_{f},b_{c}$ and $b_{o}$ are bias vectors. Layers’ notation has been omitted for clarity.

Networks using LSTM cells have offered better performance than standard recurrent units in speech recognition, where they gave state-of-the-art results in phoneme recognition [20].

### 2.2. Feature Learning with Convolutional Networks

Neural networks, whether recurrent or feedforward, can receive as input raw sensor signals. However, applying them to features derived from the raw sensor signals often leads to higher performance [21]. Discovering adequate features requires expert knowledge, which necessarily limits a systematic exploration of the feature space [12]. Convolutional networks (CNNs) have been suggested to address this [17]. A CNN with a single layer extracts features from the input signal through a convolution operation of the signal with a filter (or kernel). In a CNN, the activation of a unit represents the result of the convolution of the kernel with the input signal. By computing the activation of a unit on different regions of the same input (using a convolutional operation), it is possible to detect patterns captured by the kernels, regardless of where the pattern occurs. In CNNs, the kernels are optimised as part of the supervised training process, in an attempt to maximize the activation level of kernels for subsets of classes. A feature map is an array of units (or layer) whose units share the same parameterization (weight vector and bias). Their activation yields the result of the convolution of the kernel across the entire input data.

**Figure 2:**
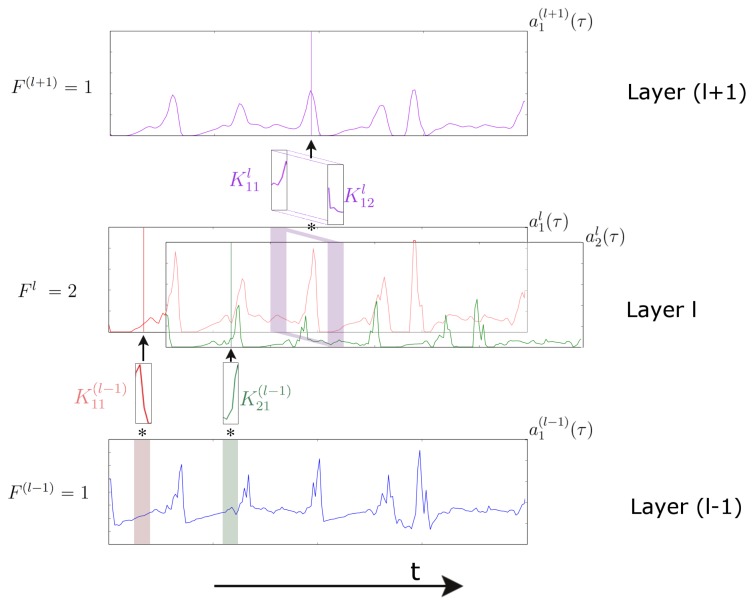
Representation of a temporal convolution over a single sensor channel in a three-layer convolutional neural network (CNN). Layer (l-1) defines the sensor data at the input. The next layer (*l*) is composed of two feature maps ($a_{1}^{l}\left(\tau \right)$ and $a_{2}^{l}\left(\tau \right)$) extracted by two different kernels ($K_{11}^{\left(l-1\right)}$ and $K_{21}^{\left(l-1\right)}$). The deepest layer (layer (l+1)) is composed by a single feature map, resulting from temporal convolution in layer *l* of a two-dimensional kernel $K_{1}^{l}$. The time axis (which is convolved over) is horizontal.

The application of the convolution operator depends on the input dimensionality. With a temporal sequence of 2D images (e.g., a video), often 2D kernels are used in a 2D spatial convolution [22]. With a one-dimensional temporal sequence (e.g., a sensor signal), often a 1D kernel is used in a temporal convolution [23]. In the 1D domain, a kernel can be viewed as a filter, capable of removing outliers, filtering the data or acting as a feature detector, defined to respond maximally to specific temporal sequences within the timespan of the kernel. Formally, extracting a feature map using a one-dimensional convolution operation is given by:

(9) $$a_{j}^{\left(l+1\right)}\left(\tau \right)=\sigma \left(b_{j}^{l}+\sum_{f=1}^{F^{l}}K_{jf}^{l}\left(\tau \right)✻a_{f}^{l}\left(\tau \right)\right)=\sigma \left(b_{j}^{l}+\sum_{f=1}^{F^{l}}\left[\sum_{p=1}^{P^{l}}K_{jf}^{l}\left(p\right)a_{f}^{l}\left(\tau -p\right)\right]\right)$$

where $a_{j}^{l}\left(\tau \right)$ denotes the feature map *j* in layer *l*, *σ* is a non-linear function, $F^{l}$ is the number of feature maps in layer *l*, $K_{jf}^{l}$ is the kernel convolved over feature map *f* in layer *l* to create the feature map *j* in layer (l+1), $P^{l}$ is the length of kernels in layer *l* and $b^{l}$ is a bias vector. When processing sensor data, this computation is applied to each sensor channel at the input independently, as shown in Figure 2; hence, the number of feature maps at the input level is $F^{1}=1$. In subsequent layers, the number of feature maps will be defined by the number of kernels within that layer (see Figure 2).

A kernel whose weights would be able to capture a specific salient pattern of a gesture would act as a feature detector. A model with several convolutional layers, in a stacked configuration where the output of layer l-1 is the input for the upper layer *l*, may be able to learn a hierarchical representation of the data, where deeper layers progressively represent the inputs in a more abstract way. Deep CNNs have had a major impact on fields, like content recommendation [24], speech recognition [25] and in computer vision [26,27,28], where they have become the *de facto* standard approach.

### 2.3. Application of Deep Networks for HAR

Deep networks are able to compute more complex transformations of the input than those networks defined by a small number or just a single hidden layer (shallow networks), offering a higher representational power.

DNNs have been applied to the wearable HAR domain, using network architectures where convolutional and non-recurrent layers are combined [17,23]. Raw signals obtained from wearable sensors were processed by convolutional layers to capture features, which were unified by dense layers to obtain a probability distribution over different human activities. Experiments on several benchmark datasets (the OPPORTUNITY, Skoda and Actitracker datasets) proved convolutional operators capable of capturing temporal signal structure within the kernel window. Results showed that these network architectures offer a model with more discriminative power, outperforming state-of-the-art approaches.

Further improvements in terms of time series classification have been obtained in the speech recognition domain, by combining convolutional and recurrent layers in a unified deep framework, which contains either standard recurrent units [29] or LSTM cells [16]. Results on different datasets, such as the TIMITphone recognition database, proved these architectures to offer a feature representation that is more easily separable and able to capture information even at different data resolutions.

The case of activity recognition in video is one of the closest problems to the HAR scenario addressed in this paper, since video data analysis can be seen as time series modelling. In the video domain, CNNs and LSTMs were shown to be suitable to combine temporal information in subsequent video frames to enable better video classification. Results of a comparative analysis on the Sports-1Mand UCF-101 datasets showed that LSTM cells were necessary to take full advantage of the motion information contained in the video and yielded the highest reported performance [30].

Several deep recurrent approaches for video gesture recognition were compared on the Montalbano dataset [22]. Models that included recurrence and convolutions improved frame-wise gesture recognition significantly. Results proved how recurrent approaches are able to capture temporal information, which provides a more discriminative data representation. It allowed outperforming non-recurrent networks and segmenting more accurately the beginning and ending frames of gestures.

Other network topologies, such as the deep belief networks (DBN), have been also applied to the activity recognition domain. DBNs are a form of generative deep learning networks, whose hidden layers are trained in a greedy layer-wise fashion. They can be used to extract a deep hierarchical representation of the training data [31]. Results on three wearable HAR datasets (OPPORTUNITY, Skoda and Darmstadt Daily Routines datasets) show how they offer a feature extraction framework with general applicability in HAR applications [32].

These related work illustrate the potential of using deep CNNs to learn features in time series and also show that LSTMs are suitable to learn temporal dynamics in sensor signals. While some work applied CNNs to activity recognition, the effective combination of convolutional and recurrent layers, which has already offered state-of-the-art results in other time series domains, such as speech recognition, has not yet been investigated in the HAR domain.

## 3. Architecture

**Figure 3:**
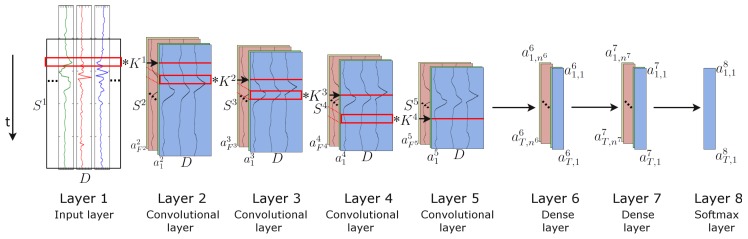
Architecture of the DeepConvLSTM (Conv, convolutional) framework for activity recognition. From the left, the signals coming from the wearable sensors are processed by four convolutional layers, which allow learning features from the data. Two dense layers then perform a non-linear transformation, which yields the classification outcome with a softmax logistic regression output layer on the right. Input at Layer 1 corresponds to sensor data of size $D\times S^{1}$, where *D* denotes the number of sensor channels and $S^{l}$ the length of features maps in layer *l*. Layers 2–5 are convolutional layers. $K^{l}$ denotes the kernels in layer *l* (depicted as red squares). $F^{l}$ denotes the number of feature maps in layer *l*. In convolutional layers, $a_{i}^{l}$ denotes the activation that defines the feature map *i* in layer *l*. Layers 6 and 7 are dense layers. In dense layers, $a_{t,i}^{l}$ denotes the activation of the unit *i* in hidden layer *l* at time *t*. The time axis is vertical.

We introduce a new DNN framework for wearable activity recognition, which we refer to as DeepConvLSTM. This architecture combines convolutional and recurrent layers. The convolutional layers act as feature extractors and provide abstract representations of the input sensor data in feature maps. The recurrent layers model the temporal dynamics of the activation of the feature maps. In this framework, convolutional layers do not include a pooling operation (see Section 5.4 for a discussion of this choice). In order to characterise the benefits brought about by DeepConvLSTM, we compare it to a “baseline” non-recurrent deep CNN. Both approaches are defined according to the network structure depicted in Figure 3. For comparison purposes, they share the same architecture, with four convolutional layers and three dense layers. The input is processed in a layer-wise format, where each layer provides the representation of the input that will be used as data for the next layer. The number of kernels in the convolutional layers and the processing units in the dense layers is the same for both cases. The main difference between DeepConvLSTM and the baseline CNN is the topology of the dense layers. In the case of DeepConvLSTM, the units of these layers are LSTM recurrent cells, and in the case of the baseline model, the units are non-recurrent and fully connected. Therefore, performance differences between the models are a product of the architectural differences and not due to better optimisation, preprocessing or *ad hoc* customisation.

The input to the network consists of a data sequence. The sequence is a short time series extracted from the sensor data using a sliding window approach (see Section 4.2 for details) composed of several sensor channels. The number of sensor channels is denoted as *D*. Within that sequence, all channels have the same number of samples $S^{1}$. The length of feature maps $S^{l}$ varies in different convolutional layers. The convolution is only computed where the input and the kernel fully overlap. Thus, the length of a feature map is defined by:

(10) $$S^{\left(l+1\right)}=S^{l}-P^{l}+1$$

where $P^{l}$ is the length of kernels in layer *l*. The length of the kernels is the same for every convolutional layer, being defined as $P^{l}=5,\forall l=2,\cdots ,5$.

### 3.1. DeepConvLSTM

DeepConvLSTM is a DNN, which comprises convolutional, recurrent and softmax layers. Firstly, sensor data are transformed through four convolutional operations, as defined in Equation (9). Convolutional layers process the input only along the axis representing time. The number of sensor channels is the same for every feature map in all layers. In Figure 3, convolution operators are displayed as ‘✻’, which is applied to a kernel whose size is delineated by the red rectangles. These convolutional layers employ rectified linear units (ReLUs) to compute the feature maps, whose non-linear function in Equation (9) is defined as σ(x)=max(0,x). Layers 6 and 7 are recurrent dense layers. The choice of the number of recurrent layers is made following the results presented in [33], where the authors showed that a depth of at least two recurrent layers is beneficial when processing sequential data. Recurrent dense layers adapt their internal state after each time step. Here, the inputs of Layer 6 at time *t* are the elements of all of the feature maps at Layer 5 at time *t*, with t=1⋯T and $T=S^{5}$. The activation of the recurrent units is computed using the hyperbolic tangent function. The output of the model is obtained from a softmax layer (a dense layer with a softmax activation function), yielding a class probability distribution for every single time step *t*. Following the notation in [22], the shorthand description of this model is: C(64)-C(64)-C(64)-C(64)-R(128)-R(128)-Sm, where $C(F^{l})$ denotes a convolutional layer *l* with $F^{l}$ feature maps, $R(n^{l})$ a recurrent LSTM layer with $n^{l}$ cells and Sm a softmax classifier.

### 3.2. Baseline Deep CNN

The baseline model is a deep CNN, which comprises convolutional, non-recurrent and softmax layers. This approach shares the convolutional layers of DeepConvLSTM. It receives the same input, a $D\times S^{1}$ sensor data sequence, and the features maps are extracted in the same way as in the DeepConvLSTM architecture. In this model, Layers 6 and 7 are non-recurrent dense layers, identical to those employed in MLPs. The activation of each unit in the first dense layer is computed using all of the feature maps from the last convolutional layer, with Equation (1). The units in the dense layers are ReLUs, with σ(x)=max(0,x). The output of the model is obtained from a softmax layer (a dense layer with a softmax activation function), yielding a probability distribution over classes. For this model, the shorthand description is: C(64)-C(64)-C(64)-C(64)-D(128)-D(128)-Sm, where $C(F^{l})$ denotes a convolutional layer *l* with $F^{l}$ feature maps, $D(n^{l})$ a dense layer with $n^{l}$ units and Sm a softmax classifier.

### 3.3. Model Implementation and Training

The neural networks here described are implemented in Theano using Lasagne [34], a lightweight library to build and train neural networks. The model training and classification are run on a GPU with 1664 cores, 1050 MHz clock speed and 4 GB RAM.

Models are trained in a fully-supervised way, backpropagating the gradients from the softmax layer through to the convolutional layers. The network parameters are optimized by minimizing the cross-entropy loss function using mini-batch gradient descent with the RMSProp update rule [35]. After experimenting with multiple per-parameter learning rate updates, we found that RMSProp consistently offered the best results with the widest tolerance to the learning rate setting. The number of parameters to optimize in a DNN varies according to the type of layers it comprises and has great impact in the time and computer power required to train the networks. The number and size of the parameters in the networks presented in Section 3.1 and Section 3.2 are detailed in Table 1.

**Table 1:** Number and size of parameters for the DeepConvLSTM architecture and for the baseline model. The final number of parameters depends on the number of classes in the classification task, denoted as $n_{c}$.

Layer	DeepConvLSTM		Baseline CNN	
	Size Per Parameter	Size Per Layer	Size Per Parameter	Size Per Layer
2	*K*: 64×5	384	*K*: 64×5	384
	b: 64		b: 64	
3–5	*K*: 64×64×5	20,544	*K*: 64×64×5	20,544
	b: 64		b: 64	
6	$W_{ai},W_{af},W_{ac},W_{ao}$: 7232×128	942,592	*W*: 57,856×128	7,405,696
	$W_{hi},W_{hf},W_{hc},W_{ho}$: 128×128		b: 128	
	$b_{i},b_{f},b_{c},b_{o}$: 128			
	$W_{ci},W_{cf},W_{co}$: 128			
	c: 128			
	h: 128			
7	$W_{ai},W_{af},W_{ac},W_{ao}$: 128×128	33,280	*W*: 128×128	16,512
	$W_{hi},W_{hf},W_{hc},W_{ho}$: 128×128		b: 128	
	$b_{i},b_{f},b_{c},b_{o}$: 128			
	$W_{ci},W_{cf},W_{co}$: 128			
	c: 128			
	h: 128			
8	*W*: $128\times n_{c}$	$\left(128\times n_{c}\right)+n_{c}$	*W*: $128\times n_{c}$	$\left(128\times n_{c}\right)+n_{c}$
	b: $n_{c}$		b: $n_{c}$	
**Total**	**996,800 $+\left(128\times n_{c}\right)+n_{c}$**		**$7,443,136+\left(128\times n_{c}\right)+n_{c}$**	

For the sake of efficiency, when training and testing, data are segmented on mini-batches of a size of 100 data segments. Using this configuration, an accumulated gradient for the parameters is computed after every mini-batch. Both models are trained with a learning rate of $10e^{-3}$ and a decay factor of ρ=0.9. Weights are randomly orthogonally initialized. We introduce a drop-out operator on the inputs of every dense layer, as a form of regularization. This operator sets the activation of randomly-selected units during training to zero with probability p=0.5.

## 4. Experimental Setup

We evaluate DeepConvLSTM on two human activity recognition datasets and compare the performance against the baseline CNN, which provides a performance reference for deep networks, and against results reported in the literature on these datasets using other machine learning techniques.

### 4.1. Benchmark Datasets

Human activities can be defined as periodic, such as walking and bicycling, static, such as being seated and standing still, or sporadic, such as goal-oriented gestures (e.g., drinking from a cup) [8]. Benchmarking of activity recognition must be conducted on datasets comprising a variety of these types of activities. Furthermore, human activities (*i.e.*, goal-oriented gestures, such as “fetching a cup”) are often embedded in a large *Null* class (the *Null* class corresponds to the time spans that do not cover “interesting” activities, such as, e.g., when a user is not engaging in one of the activities that is relevant to the scenario at hand). The recognition of activities embedded in a *Null* class tends to be more challenging, as the recognition system must implicitly identify the start and end point of data comprising a gesture and then classify it. A number of datasets have been published for activity recognition, including the OPPORTUNITY [36], PAMAP [37], Skoda [38] and mHealth [39] datasets. In this paper, we selected two datasets for the evaluation of our approach based on the variety and variability of activities and their presence in the HAR literature.

#### 4.1.1. The OpportunityDataset

The OPPORTUNITY dataset [36] comprises a set of complex naturalistic activities collected in a sensor-rich environment. Overall, it contains recordings of four subjects in a daily living scenario performing morning activities, with sensors of different modalities integrated in the environment, in objects and on the body. During the recordings, each subject performed a session five times with activities of daily living (ADL) and one drill session. During each ADL session, subjects perform the activities without any restriction, by following a loose description of the overall actions to perform (*i.e.*, checking ingredients and utensils in the kitchen, preparing and drinking a coffee, preparing and eating a sandwich, cleaning up). During the drill sessions, subjects performed 20 repetitions of a predefined sorted set of 17 activities. The dataset contains about 6 hours of recordings in total.

The OPPORTUNITY dataset comprises both static/periodic and sporadic activities. It is available on the UCIMachine Learning repository and has been used by numerous third party publications (e.g., [23,32,40]). Most importantly, it has been used in an open activity recognition challenge where participants (listed in Table 3) competed to achieve the highest performance on the recognition of modes of locomotion, as well as sporadic gestures [7]. This dataset is publicly available and can be downloaded from [41].

For this paper, we have used the same subset employed in the OPPORTUNITY challenge to train and test our models. We train the models on the data of all ADL and drill sessions for the first subject and on ADL1, ADL2 and drill sessions for Subjects 2 and 3. We report classification performance on a testing set composed of ADL4 and ADL5 for Subjects 2 and 3. ADL3 datasets for Subjects 2 and 3 were left for validation.

**Figure 4:**
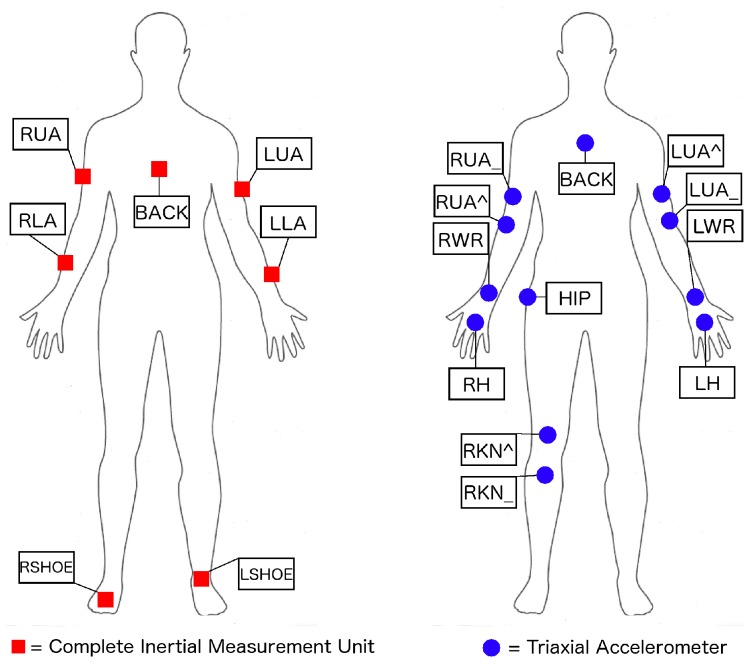
Placement of on-body sensors used in the OPPORTUNITYdataset (left: inertial measurements units; right: 3-axis accelerometers) [7].

In terms of the sensor setting, we follow the OPPORTUNITY challenge guidelines, taking into account only the on-body sensors. This includes 5 commercial RS485-networked XSense inertial measurement units (IMU) included in a custom-made motion jacket, 2 commercial InertiaCube3 inertial sensors located on each foot (Figure 4, left) and 12 Bluetooth acceleration sensors on the limbs (Figure 4, right). Each IMU is composed of a 3D accelerometer, a 3D gyroscope and a 3D magnetic sensor, offering multimodal sensor information. Each sensor axis is treated as an individual channel yielding an input space with a dimension of 113 channels. The sample rate of these sensors is 30 Hz.

In this study, sensor data were pre-processed to fill in missing values using linear interpolation and to do a per channel normalization to interval [0,1].

The OPPORTUNITY dataset includes several annotations of gestures and modes of locomotion/postures. In this paper, we have focused the models on two tasks defined in the OPPORTUNITY challenge:

- Task A: recognition modes of locomotion and postures. The goal of this task is to classify modes of locomotion from the full set of body-worn sensors. This is a 5-class segmentation and classification problem.
- Task B: recognition of sporadic gestures. This task concerns recognition of the different right-arm gestures. This is an 18-class segmentation and classification problem.

The activities included in the dataset for each task are summarised in Table 2.

**Table 2:** Class labels for the OPPORTUNITY and Skoda datasets. The OPPORTUNITY dataset is divided into activities belonging to Task A (modes of locomotion) and Task B (gesture recognition). For each class, we report the number of times an activity is performed and the number of instances obtained by the sliding window (all subjects combined). The *Null* class corresponds to the time intervals where there are no activities of interest.

OPPORTUNITY						Skoda		
Gestures			Modes of Locomotion					
Name	# of Repetitions	# of Instances	Name	# of Repetitions	# of Instances	Name	# of Repetitions	# of Instances
Open Door 1	94	1583	Stand	1267	38,429	Write on Notepad	58	20,874
Open Door 2	92	1685	Walk	1291	22,522	Open Hood	68	24,444
Close Door 1	89	1497	Sit	124	16,162	Close Hood	66	23,530
Close Door 2	90	1588	Lie	30	2866	Check Gaps Door	67	16,961
Open Fridge	157	196	*Null*	283	16,688	Open Door	69	10,410
Close Fridge	159	1728				Check Steering Wheel	69	12,994
Open Dishwasher	102	1314				Open and Close Trunk	63	23,061
Close Dishwasher	99	1214				Close both Doors	69	18,039
Open Drawer 1	96	897				Close Door	70	9783
Close Drawer 1	95	781				Check Trunk	64	19,757
Open Drawer 2	91	861						
Close Drawer 2	90	754						
Open Drawer 3	102	1082						
Close Drawer 3	103	1070						
Clean Table	79	1717						
Drink from Cup	213	6115						
Toggle Switch	156	1257						
*Null*	1605	69,558						

#### 4.1.2. The Skoda Dataset

The Skoda Mini Checkpoint dataset [38] describes the activities of assembly-line workers in a car production environment. These gestures are similar to those performed at the quality assurance checkpoint of a production plant and are listed in Table 2.

In the study, one subject wore 20 3D accelerometers on both arms. We restrict our experiments to the 10 sensors placed on the right arm. The original sample rate of this dataset was 98 Hz, but it was decimated to 30 Hz for comparison purposes with the OPPORTUNITY dataset. The dataset contains 10 manipulative gestures. The recording is about 3 h long, comprising about 70 repetitions per gesture. This dataset is publicly available and can be downloaded from [42]. The Skoda dataset has been employed to evaluate decision fusion techniques in sensor networks [38] and deep learning techniques [23,43], which makes it a suitable dataset to evaluate our proposed solution.

### 4.2. Performance Measure

The OPPORTUNITY and Skoda datasets were recorded continuously. We use a sliding window of fixed length to segment the data. We refer to each window as a “sequence”, which is the input of the network. Following the OPPORTUNITY challenge experimental setup, the length of the window is 500 ms, with a step size of 250 ms. The number of instances (segments) obtained after using this sliding window configuration is detailed per dataset in Table 2.

**Figure 5:**
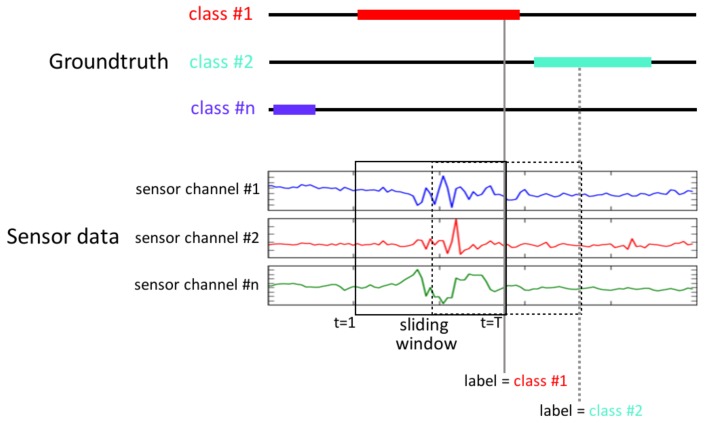
Sequence labelling after segmenting the data with a sliding window. The sensor signals are segmented by a jumping window. The activity class within each sequence is considered to be the ground truth label annotated at the sample *T* of that window.

The class associated with each segment corresponds to the gesture that has been observed during that interval. Given a sliding window of length *T*, we choose the class of the sequence as the label at t=T, or in other words, the label of the last sample in the window, as depicted in Figure 5.

**Figure 6:**
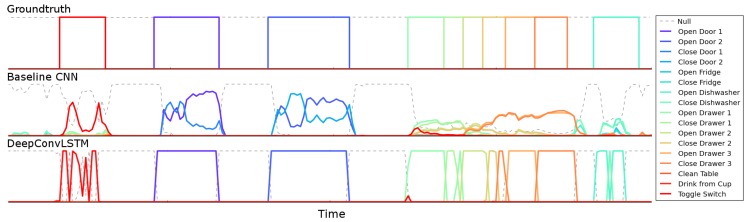
Output class probabilities for a ~25 s-long fragment of sensor signals in the test set of the OPPORTUNITY dataset, which comprises 10 annotated gestures. Each point in the plot represents the class probabilities obtained from processing the data within a sequence of 500 ms obtained from a sliding window ending at that point. The dashed line represents the *Null* class. DeepConvLSTM offers a better performance identifying the start and ending of gestures.

As stated in Section 3, DeepConvLSTM outputs a class probability distribution for every single time step *t* in the sequence (*i.e*., the 500-ms window of the sensor signal). However, we are interested in the class probability distribution once DeepConvLSTM has observed the entire 500-ms sequence. Several approaches exist for this [30]: (1) using the prediction at the last time step *T*; (2) max-pooling the predictions over the sequence; (3) summing all of the sequence predictions over time and returning the most frequent. Since the memory of LSTM units tends to become progressively more informed as a function of the number of samples they have seen, DeepConvLSTM returns the class probability distribution only at the last time step *T*, when the full sequence has been observed. Thus, at the time of each sample of the original sensor signal, DeepConvLSTM provides a class probability distribution inferred from processing a 500-ms extract of the sensor signal prior to that time, as illustrated in Figure 6. In terms of comparison, that value is also the most relevant, given that the sample at time *T* is the one defining the label of the sequence in the ground truth.

Naturalistic human activity datasets are often highly unbalanced. Class imbalance occurs when some classes are represented by a large number of examples while others are represented by only a few [44]. The gesture recognition task of the OPPORTUNITY dataset is extremely imbalanced, as the *Null* class represents more than 75% of the recorded data (76%, 82% and 76% for Subjects 1–3, respectively). The overall classification accuracy is not an appropriate measure of performance, since a trivial classifier that predicted every instance as the majority class could achieve very high accuracy. Therefore, we evaluate the models using the F-measure ($F_{1}$), a measure that considers the correct classification of each class equally important. The $F_{1}$ score combines two measures defined in terms of the total number of correctly-recognized samples and which are known in the information retrieval community as precision and recall. Precision is defined as $\frac{TP}{TP+FP}$, and recall corresponds to $\frac{TP}{TP+FN}$, where TP, FP are the number of true and false positives, respectively, and FN corresponds to the number of false negatives. Class imbalance is countered by weighting classes according to their sample proportion:

(11) $$F_{1}=\sum_{i}2*w_{i}\frac{precision_{i}\cdot recall_{i}}{precision_{i}+recall_{i}}$$

where *i* is the class index and $w_{i}=n_{i}/N$ is the proportion of samples of class *i*, with $n_{i}$ being the number of samples of the *i*-th class and *N* being the total number of samples.

## 5. Results and Discussion

In this section, we present the results and discuss the outcome. We show the performance of the approaches and also evaluate some of their key parameters to obtain some insights about the suitability of these approaches for the domain.

### 5.1. Performance Comparison

The results of the proposed deep methods on the OPPORTUNITY dataset and the Skoda dataset are shown in Table 4 and Table 9, respectively. In the case of the OPPORTUNITY dataset, we report here the classification performance either including or ignoring the *Null* class. Including the *Null* class may lead to an overestimation of the performance given its large prevalence. By providing both results, we get better insights about the type of errors made by the models.

Table 3 includes a comprehensive list of past published classification techniques employed on the datasets. The techniques competing in the OPPORTUNITY challenge were sliding window based and only differ in the classifier and features extracted.

**Table 3:** Baseline classifiers included in the datasets’ comparative evaluation.

OPPORTUNITY Dataset		
	**Challenge Submissions [7]**	
	**Method**	**Description**
	LDA	Linear discriminant analysis. Gaussian classifier that classifies on the assumption that the features are normally distributed and all classes have the same covariance matrix.
	QDA	Quadratic discriminant analysis. Similar to the LDA, this technique also assumes a normal distribution for the features, but the class covariances may differ.
	NCC	Nearest centroid classifier. The Euclidean distance between the test sample and the centroid for each class of samples is used for the classification.
	1NN	k nearest neighbour algorithm. Lazy algorithm where the Euclidean distances between a test sample and the training samples are computed and the most frequently-occurring label of the *k*-closest samples is the output.
	3NN	See 1NN. Using 3 neighbours.
	UP	Submission to the OPPORTUNITY challenge from U. of Parma. Pattern comparison using mean, variance, maximum and minimum values.
	NStar	Submission to the OPPORTUNITY challenge from U. of Singapore. kNN algorithm using a single neighbour and normalized data.
	SStar	Submission to the OPPORTUNITY challenge from U. of Singapore. Support vector machine algorithm using scaled data.
	CStar	Submission to the OPPORTUNITY challenge from U. of Singapore. Fusion of a kNN algorithm using the closest neighbour and a support vector machine.
	NU	Submission to the OPPORTUNITY challenge from U. of Nagoya. C4.5 decision tree algorithm using mean, variance and energy.
	MU	Submission to the OPPORTUNITY challenge from U. of Monash. Decision tree grafting algorithm.
	**Deep approaches**	
	Method	Description
	CNN [17]	Results reported by Yang *et. al.*, in [17]. The value is computed using the average performance for Subjects 1, 2 and 3.
**Skoda dataset**		
	**Deep approaches**	
	Method	Description
	CNN [23]	Results reported by Ming Zeng *et. al.*, in [23]. Performance computed using one accelerometer on the right arm to identify all activities.
	CNN [43]	Results reported by Alsheikh *et. al.*, in [43]. Performance computed using one accelerometer node (id #16) to identify all activities.

**Table 4:** $F_{1}$ score performance on OPPORTUNITY dataset for the gestures and modes of locomotion recognition tasks, either including or ignoring the *Null* class. The best results are highlighted in bold.

Method	Modes of Locomotion	Modes of Locomotion	Gesture Recognition	Gesture Recognition
	(No *Null* Class)		(No *Null* Class)	
	**OPPORTUNITY Challenge Submissions**			
LDA	0.64	0.59	0.25	0.69
QDA	0.77	0.68	0.24	0.53
NCC	0.60	0.54	0.19	0.51
1 NN	0.85	0.84	0.55	0.87
3 NN	0.85	0.85	0.56	0.85
UP	0.84	0.60	0.22	0.64
NStar	0.86	0.61	0.65	0.84
SStar	0.86	0.64	0.70	0.86
CStar	0.87	0.63	0.77	0.88
NU	0.75	0.53		
MU	0.87	0.62		
	Deep architectures			
CNN [17]				0.851
Baseline CNN	0.912	0.878	0.783	0.883
DeepConvLSTM	**0.930**	**0.895**	**0.866**	**0.915**

From the results in Table 4, we can see that DeepConvLSTM consistently outperforms baselines on both tasks. When compared to the best submissions of the OPPORTUNITY challenge, it improves the performance by 6% on average. For some specific tasks, it can be noticed how DeepConvLSTM offers a striking performance improvement: there is more than a 9% improvement in the gesture recognition task without the *Null* class when compared to the OPPORTUNITY challenge models. DeepConvLSTM also improves by 6% over results previously reported by Yang *et. al.* [17] using a CNN.

The baseline CNN also offers better results than the OPPORTUNITY submissions in the recognition of models of locomotion. However, in the case of gesture recognition, it obtains a similar recognition performance as the ensemble approach named CStar. These results of the baseline CNN are consistent with those obtained previously by Yang *et. al.* in [17] using a CNN on raw signal data. Among deep architectures, DeepConvLSTM systematically performs better than the CNNs, improving the performance by 5% on average on the OPPORTUNITY dataset.

In Figure 6, we illustrate the differences in the output predictions of the different architectures on the OPPORTUNITY dataset. One of the main challenges in this domain is the automatic segmentation of the activities. The baseline CNN approach tends to make more mistakes and has difficulties making crisp decisions about the boundaries of the gestures. It has troubles defining where the gesture starts or ends.

The confusion matrices on the OPPORTUNITY dataset for the gesture recognition task are illustrated in Table 5 and Table 7 for the DeepConvLSTM approach and in Table 6 and Table 8 for the baseline CNN. The confusion matrices contain information about actual and predicted gesture classifications done by the system, to identify the nature of the classification errors, as well as their quantities. Each cell in the confusion matrix represents the number of times that the gesture in the row is classified as the gesture in the column. Given the class imbalance in the data due to the presence of the dominant *Null* class, we report confusion matrices including and ignoring the *Null* class, in order to get better insights on the actual system performance.

When the *Null* class is included in the recognition task (see Table 5 and Table 6), most classification errors, both false positives and false negatives, are related to this class. This is the most realistic setup, where almost 75% of the data (see Table 2) processed is not considered as an activity of interest.

When the *Null* class is removed from the classification task (see Table 7 and Table 8), both approaches tend to misclassify gestures that are relatively similar, such as “Open Door 2”-“Close Door 2” or “Open Fridge”-“Close Fridge”. This may be because these gestures involve the activation of the same type of sensors, but with a different sequentiality. In the case of gestures “Open Door 2”-“Close Door 2”, one is misclassified as the other 44 times by the baseline CNN, while DeepConvLSTM made only 14 errors. Similarly, for gestures “Open Drawer 3”-“Close Drawer 3”, the baseline CNN made 33 errors, while DeepConvLSTM misclassified only 14 sequences. The better performance of DeepConvLSTM for these similar gestures may be explained by the ability of LSTM cells to capture temporal dynamics within the data sequence processed. On the other hand, the baseline CNN is only capable of modelling time sequences up to the length of the kernels.

**Table 5:** Confusion matrix for OPPORTUNITY dataset using DeepConvLSTM.

		Predicted Gesture																	
		Null	Open Door 1	Open Door 2	Close Door 1	Close Door 2	Open Fridge	Close Fridge	Open Dishwasher	Close Dishwasher	Open Drawer 1	Close Drawer 1	Open Drawer 2	Close Drawer 2	Open Drawer 3	Close Drawer 3	Clean Table	Drink from Cup	Toggle Switch
**Actual Gesture**	*Null*	**13,532**	16	5	15	13	54	35	35	72	10	13	5	4	22	39	7	158	29
	Open Door 1	10	**76**	0	10	0	0	0	0	0	0	0	0	0	0	0	0	0	0
	Open Door 2	7	0	**155**	0	2	0	0	0	0	0	0	0	0	0	0	0	0	0
	Close Door 1	8	15	0	**78**	0	0	0	0	0	0	0	0	0	0	0	0	0	0
	Close Door 2	10	0	0	0	**130**	0	0	0	0	0	0	0	0	0	0	0	0	0
	Open Fridge	111	0	0	0	0	**253**	22	2	0	0	0	0	0	0	0	0	0	1
	Close Fridge	41	0	0	0	0	19	**210**	0	1	0	0	0	0	0	0	0	0	0
	Open Dishwasher	61	0	0	0	0	6	0	**99**	4	1	0	0	0	0	0	0	0	0
	Close Dishwasher	43	0	0	0	0	2	0	10	**79**	0	0	0	1	0	0	0	0	0
	Open Drawer 1	10	0	0	0	0	0	0	3	1	**38**	6	2	1	3	1	0	0	1
	Close Drawer 1	20	0	0	0	0	1	0	0	0	8	**46**	0	0	0	0	0	0	0
	Open Drawer 2	13	0	0	0	0	0	0	0	1	18	2	**29**	6	1	0	0	0	1
	Close Drawer 2	5	0	0	0	0	0	0	0	2	1	5	4	**25**	0	3	0	0	0
	Open Drawer 3	14	0	0	0	0	0	0	0	0	0	0	8	0	**88**	3	0	0	0
	Close Drawer 3	6	0	0	0	0	0	0	0	0	0	0	2	9	5	**80**	0	0	0
	Clean Table	88	0	0	0	0	0	0	0	0	0	0	0	0	0	0	**81**	2	0
	Drink from Cup	143	1	0	0	0	0	0	1	1	0	0	0	0	0	0	0	**397**	0
	Toggle Switch	57	0	0	0	0	0	0	0	0	2	2	0	0	0	0	0	0	**122**

**Table 6:** Confusion matrix for OPPORTUNITY dataset using the baseline CNN.

		Predicted Gesture																	
		*Null*	Open Door 1	Open Door 2	Close Door 1	Close Door 2	Open Fridge	Close Fridge	Open Dishwasher	Close Dishwasher	Open Drawer 1	Close Drawer 1	Open Drawer 2	Close Drawer 2	Open Drawer 3	Close Drawer 3	Clean Table	Drink from Cup	Toggle Switch
**Actual Gesture**	*Null*	**13,752**	5	8	6	5	39	18	14	29	2	0	1	1	40	20	2	114	8
	Open Door 1	17	**51**	0	28	0	0	0	0	0	0	0	0	0	0	0	0	0	0
	Open Door 2	15	0	**111**	0	38	0	0	0	0	0	0	0	0	0	0	0	0	0
	Close Door 1	10	22	0	**69**	0	0	0	0	0	0	0	0	0	0	0	0	0	0
	Close Door 2	9	0	7	0	**124**	0	0	0	0	0	0	0	0	0	0	0	0	0
	Open Fridge	130	0	0	0	0	**220**	34	4	1	0	0	0	0	0	0	0	0	0
	Close Fridge	49	0	0	0	0	76	**146**	0	0	0	0	0	0	0	0	0	0	0
	Open Dishwasher	108	0	0	0	0	4	0	**45**	14	0	0	0	0	0	0	0	0	0
	Close Dishwasher	75	0	0	0	0	4	0	30	**26**	0	0	0	0	0	0	0	0	0
	Open Drawer 1	31	0	0	0	0	0	0	0	0	**27**	5	0	0	2	0	0	0	1
	Close Drawer 1	40	0	0	0	0	0	0	0	0	19	**16**	0	0	0	0	0	0	0
	Open Drawer 2	36	0	0	0	0	0	0	0	0	9	1	**18**	1	6	0	0	0	0
	Close Drawer 2	14	0	0	0	0	0	0	0	0	3	1	13	**5**	9	0	0	0	0
	Open Drawer 3	29	0	0	0	0	0	0	0	0	0	0	0	0	**56**	28	0	0	0
	Close Drawer 3	9	0	0	0	0	0	0	0	0	0	0	0	0	51	**42**	0	0	0
	Clean Table	98	0	0	0	0	0	0	0	0	0	0	0	0	0	0	**73**	0	0
	Drink from Cup	194	0	0	0	0	0	0	0	0	0	0	0	0	0	0	0	**349**	0
	Toggle Switch	99	0	0	0	0	0	0	0	0	2	0	0	0	0	0	0	0	**82**

**Table 7:** Confusion matrix for OPPORTUNITY dataset using DeepConvLSTM (without the *Null* class).

		Predicted Gesture																
		Open Door 1	Open Door 2	Close Door 1	Close Door 2	Open Fridge	Close Fridge	Open Dishwasher	Close Dishwasher	Open Drawer 1	Close Drawer 1	Open Drawer 2	Close Drawer 2	Open Drawer 3	Close Drawer 3	Clean Table	Drink from Cup	Toggle Switch
**Actual Gesture**	Open Door 1	**81**	0	16	0	0	0	0	0	0	0	0	0	0	0	0	0	1
	Open Door 2	0	**149**	1	12	0	0	0	0	0	0	0	0	0	0	0	0	0
	Close Door 1	15	0	**73**	0	0	0	0	0	0	0	0	0	0	0	0	0	0
	Close Door 2	0	2	1	**124**	0	0	0	0	0	0	0	0	0	0	0	0	0
	Open Fridge	1	1	0	0	**342**	29	11	2	0	1	0	0	0	0	1	1	2
	Close Fridge	0	0	0	0	10	**258**	0	1	1	2	0	0	0	0	0	1	1
	Open Dishwasher	0	0	0	0	4	0	**151**	5	1	0	0	0	0	2	0	2	2
	Close Dishwasher	0	0	0	0	4	0	15	**107**	0	0	0	2	0	6	0	1	2
	Open Drawer 1	0	0	0	0	0	0	1	2	**36**	17	0	1	0	2	0	0	8
	Close Drawer 1	0	1	0	0	1	0	0	0	5	**66**	1	0	0	0	0	0	0
	Open Drawer 2	0	0	0	0	1	0	1	0	13	8	**35**	3	1	0	0	0	5
	Close Drawer 2	0	0	0	0	1	0	0	4	2	10	3	**26**	1	0	0	0	0
	Open Drawer 3	0	0	0	0	0	0	5	0	2	0	7	4	**87**	7	0	0	1
	Close Drawer 3	0	0	0	0	0	0	0	1	0	0	0	27	7	**66**	0	0	0
	Clean Table	0	0	0	0	2	1	3	0	0	0	0	0	0	0	**147**	17	0
	Drink from Cup	1	1	2	1	0	0	24	1	0	0	0	0	0	0	1	**515**	0
	Toggle Switch	0	0	1	0	1	0	0	0	3	3	0	0	0	0	1	1	**161**

**Table 8:** Confusion matrix for OPPORTUNITY dataset using the baseline CNN (without the *Null* class).

		Predicted Gesture																
		Open Door 1	Open Door 2	Close Door 1	Close Door 2	Open Fridge	Close Fridge	Open Dishwasher	Close Dishwasher	Open Drawer 1	Close Drawer 1	Open Drawer 2	Close Drawer 2	Open Drawer 3	Close Drawer 3	Clean Table	Drink from Cup	Toggle Switch
**Actual Gesture**	Open Door 1	**73**	0	23	0	0	0	0	0	0	0	0	0	0	0	1	0	1
	Open Door 2	0	**111**	0	43	0	2	0	1	0	0	0	0	0	0	1	0	4
	Close Door 1	22	0	**63**	2	0	0	0	0	0	0	0	0	0	0	0	0	1
	Close Door 2	2	4	1	**118**	0	0	0	0	0	0	0	0	0	0	1	0	1
	Open Fridge	1	1	0	0	**304**	59	17	1	4	0	0	0	1	0	1	0	2
	Close Fridge	0	0	0	0	20	**243**	5	2	2	1	0	0	0	0	0	1	0
	Open Dishwasher	0	0	0	0	15	1	**121**	11	5	0	0	0	6	4	0	1	3
	Close Dishwasher	0	0	0	0	7	11	19	**90**	1	0	3	1	0	4	1	0	0
	Open Drawer 1	0	0	0	0	3	0	2	3	**35**	12	6	0	1	1	0	0	4
	Close Drawer 1	0	0	0	0	0	1	1	0	16	**51**	3	0	0	0	0	2	0
	Open Drawer 2	0	0	0	0	4	0	2	0	19	3	**31**	5	2	0	0	0	1
	Close Drawer 2	0	0	0	0	0	0	1	1	4	1	15	**18**	1	6	0	0	0
	Open Drawer 3	0	0	0	0	1	0	6	1	3	0	9	0	**62**	29	1	0	1
	Close Drawer 3	0	0	0	0	0	0	0	2	0	0	0	1	14	**84**	0	0	0
	Clean Table	1	0	2	0	9	11	0	1	0	0	0	0	0	0	**134**	12	0
	Drink from Cup	3	1	4	1	4	6	9	14	0	0	3	0	0	0	2	**499**	0
	Toggle Switch	0	1	1	0	0	4	0	0	15	1	0	0	0	0	0	0	**149**

From the results in Table 9, we can see that DeepConvLSTM outperforms other deep non-recurrent approaches on the Skoda dataset, improving the best reported result by 6%. The Skoda dataset has some specific characteristics: the gestures are long on average; it does not contain a *Null* class; and unlike the OPPORTUNITY dataset, it is quite well balanced. The greater length of the gestures does not diminish the performance of the model. These results corroborate our findings, supporting that the use of LSTM brings a significant advantage across very different scenarios.

**Table 9:** $F_{1}$ score performance on the Skoda dataset.

Method	
CNN [23]	0.861
CNN [43]	0.893
Baseline CNN	0.884
DeepConvLSTM	**0.958**

### 5.2. Multimodal Fusion Analysis

Wearable activity recognition can make use of a variety of sensors. While accelerometers tend to be extremely small and low power, inertial measurement units are more complex (combining accelerometers, gyroscopes and magnetic sensors), but can provide accurate limb orientation. It is therefore important for an activity recognition framework to be applicable to a wide range of commonly-used sensor modalities to accommodate for the various size and power trade-offs.

We evaluate how the automated feature extraction provided by the kernels in convolutional layers is suitable to deal with signals of sensors of different modalities. In Table 10, we show the performance of DeepConvLSTM at recognizing gestures on the OPPORTUNITY dataset (without the *Null* class) for different selections of sensors. It can be noticed how,without any specific preprocessing, convolution operations can be interchangeably applied to individual sensor modalities. Starting from a 69% $F_{1}$ score using only the accelerometers on the dataset, the performance improves on average by 15% fusing accelerometers and gyroscopes and by 20% when fusing accelerometers, gyroscopes and magnetic sensors. As the number of sensor channels is increased, the performance of the model is consistently improved, regardless of the modalities of the sensors. These results demonstrate that the convolutional layers can extract features from sensor signals of different modalities without *ad hoc* preprocessing.

**Table 10:** Performance using different sensor modalities.

	Accelerometers	Gyroscopes	Accelerometers	Accelerometers	Opportunity
			+ Gyroscopes	+ Gyroscopes	Sensors Set
				+ Magnetic	
# of sensors channels	15	15	30	45	113
$F_{1}$ score	0.689	0.611	0.745	0.839	0.864

### 5.3. Hyperparameters Evaluation

We characterise the influence of the key hyperparameters of the system. We evaluate the influence of two key architectural parameters: the sequence length processed by the network and the number of convolutional layers.

As previously stated, the input of the recurrent model is composed of a 500-ms data sequence. Therefore, the gradient signal is unable to notice time dependencies longer than the length of this sequence. Firstly we want to evaluate the influence of this parameter in the recognition performance of gestures with different durations, in particular if the gestures are significantly longer or shorter than the sequence duration.

**Figure 7:**
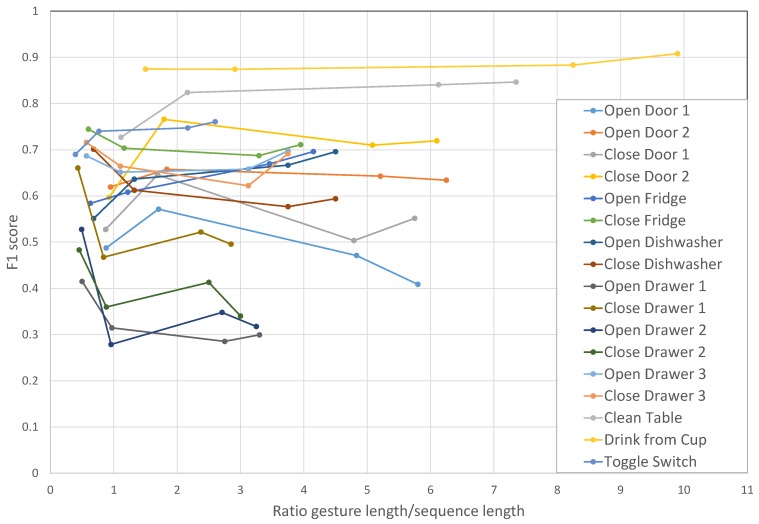
$F_{1}$ score performance of DeepConvLSTM on the OPPORTUNITY dataset. Classification performance is displayed individually per gesture, for different lengths of the input sensor data segments. Experiments carried out with sequences of length of 400 ms, 500 ms, 1400 ms and 2750 ms. The horizontal axis represents the ratio between the gesture length and the sequence length (ratios under one represent performance for gestures whose durations are shorter than the sequence duration).

The $F_{1}$ score on individual gestures of the dataset are shown in Figure 7. This figure displays performance at recognizing individual gestures as a function of the ratio between the gesture length and the sequence length. Ratios under one represent performance for gestures whose durations are shorter than the sequence duration and, thus, that can be fully observed by the network before it provides an output prediction. Besides 500 ms, we carried out experiments with sequences of lengths of 400 ms, 1400 ms and 2750 ms. For most gestures, there are no significant performance changes when modifying the length of the sequence, although shorter gestures seem to benefit from being completely included in the sequence observed by the model. That is the case for several short gestures (“Open Drawer 1”, “Close Drawer 1”, “Open Drawer 2”, “Close Drawer 2”) when their ratio is under one. When the gesture duration is longer than the sequence duration, DeepConvLSTM can only come up with a classification result based on a partial view of the temporal unfolding of the features within the sequence. However, results show that DeepConvLSTM can nevertheless obtain good performance. For example, the gesture “drink from cup”, which is 10-times longer than the sequence in one of the experiments, on average achieves a 0.9 $F_{1}$ score. We speculate that this is due to the fact that longer gestures (as “clean table” or “drink from cup” in this dataset) may be made of several shorter characteristic patterns, which allows DeepConvLSTM to spot and classify the gesture even without a complete view of it.

**Figure 8:**
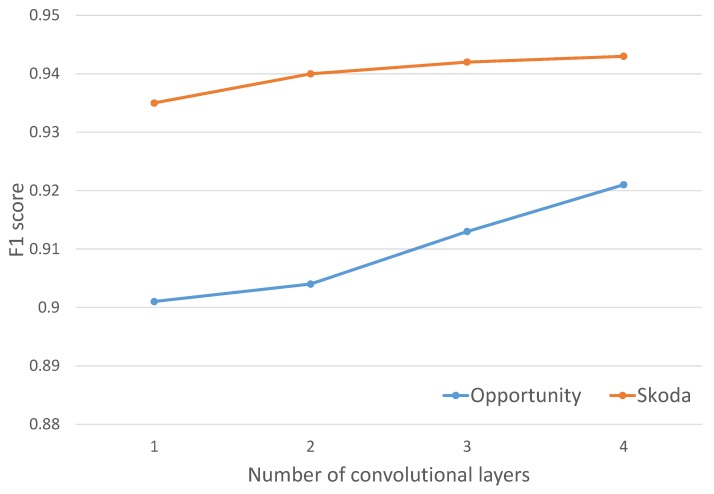
Performance of Skoda and OPPORTUNITY (recognizing gestures and with the *Null* class) datasets with different numbers of convolutional layers.

We characterised the effect of the number of convolutional layers employed to automatically learn feature representations. Figure 8 shows that increasing the number of convolutional layers tends to increase the performance for the OPPORTUNITY dataset, improving by 1% when a new layer is added. Performance changes are not significant in the case of the Skoda dataset, showing a plateau. Results for the OPPORTUNITY dataset show that performance may be further improved if the number of convolution operations are increased.

### 5.4. Discussion

The main findings from the direct comparison of our novel DeepConvLSTM against the baseline model using standard feedforward units in the dense layer is that: (i) DeepConvLSTM reaches a higher F1 score; (ii) it offers better segmentation characteristics illustrated by clearer cut decision boundaries between activities; (iii) it is significantly better able to disambiguate closely-related activities, which tend to differ only by the ordering of the time series (e.g., “Open/Close Door 2” or “Open/Close Drawer 3”); and (iv) it is applicable even if the gestures are longer than the observation window. These findings support the hypothesis that the LSTM-based model takes advantage of learning the temporal feature activation dynamics, which the baseline model is not capable of modelling.

DeepConvLSTM is and eight-layer deep network. Other publications showed much deeper networks, such as “GoogLeNet”, which is a 27-layer deep neural network applied to image classification [45]. Indeed, findings illustrated in Figure 8 show that increasing further the number of layers may be beneficial, especially for the OPPORTUNITY dataset. Training time, however, increases with the number of layers, and depending on computational resources available, future work may consider how to find trade-offs between system performance and training time. This is especially important as a deep learning approach tends to be more suitable for training on a “cloud” infrastructure, possibly using data contributed by individual wearables (e.g., as in [46]). Therefore, the choice of the number of layers is not solely a function of the desired performance, but also of the computational budget available.

In the literature, CNN frameworks often include convolutional and pooling layers successively, as a measure to reduce data complexity and introduce translation invariant features. Nevertheless, such an approach is not strictly part of the architecture, and in the time series domain, we can see some examples of CNNs where not every convolutional layer is followed by a pooling operation [16]. DeepConvLSTM does not include pooling operations because the input of the network is constrained by the sliding window mechanism defined by the OPPORTUNITY challenge, and this fact limits the possibility of downsampling the data, given that DeepConvLSTM requires a data sequence to be processed by the recurrent layers. However, without the sliding window requirement, a pooling mechanism could be useful to cover different sensor data time scales at deeper layers. With the introduction of pooling layers, it would be possible to have different convolutional layers operating on sensor data sequences downsampled at different levels.

As general guidelines, we would recommend to focus the main effort on optimizing hyperparameters related to the network architecture, which have the major influence on performance. Indeed, parameters related to the learning and regularization processes seem to have less overall influence on the performance. For instance, we tested higher drop-out rates (p>0.5) with no difference in terms of performance. These results are consistent with those presented in [23]. Nevertheless, there is a power-performance trade-off, and stacking more layers to augment the hierarchic representation of the features may not be relevant if one factors computational aspects.

We show how convolution operations are robust enough to be directly applied to raw sensor data, to learn features (salient patterns) that, within a deep framework, successfully outperformed previous results on the problem. A main benefit of using CNNs is that hand-crafted or heuristic features can be avoided, thus minimising engineering bias. This is particularly important, as activity recognition techniques are applied to domains that include more complex activities or open-ended scenarios, where classifiers must adaptively model a varying number of classes.

It is also noticeable, in terms of data, how the recurrent model is capable of obtaining a very good performance with relatively small datasets, since the largest training dataset used during the experiments (the one corresponding to the OPPORTUNITY dataset) is composed of ~80 k sensor samples, corresponding to 6 h of recordings. This seems to indicate that although deep learning techniques are often employed with large amounts of data, (e.g., millions of frames in computer vision [22]), they may actually be applicable to problem domains where acquiring annotated data is very costly, such as in supervised activity recognition.

Although LSTM cells are composed of a much higher number of parameters per cell, the overall number of parameter values is significantly larger for the baseline CNN model than for DeepConvLSTM. For the specific case of the OPPORTUNITY dataset with a *Null* class and following the equation in Table 1, the parameters of DeepConvLSTM are composed of 999,122 values, while the baseline CNN parameters contain 7,445,458 values; this represents an increase of 600%. As illustrated in Table 1, this difference in size is due to the type of connection between the convolutional and dense layers (Layers 5 and 6). In the fully-connected architecture, the units in the dense layer (Layer 6) have to be connected with every value of the last feature map (Layer 5), needing a very large weight matrix to parametrize this connection. On the other hand, the recurrent model processes the feature map sample by sample, thus requiring a much reduced number of parameter values. Although DeepConvLSTM is a more complex approach, it is composed of much smaller parameters, and this has a direct beneficial effect in the memory and computational efforts required to use this approach.

However, in terms of training and classification time, there is not such a significant difference between the two models, despite the more complex computational units included in the dense layers of DeepConvLSTM. Training DeepConvLSTM on the OPPORTUNITY dataset requires 340.3 min to converge, while the baseline CNN requires 282.2 min. The classification time of the baseline CNN is 5.43 s, while DeepConvLSTM needs 6.68 s to classify the whole dataset. On average, within a second, DeepConvLSTM can classify almost 15 min of data. Thus, this implementation is suitable for online HAR on the GPU used in this work.

We have not yet implemented DeepConvLSTM on a wearable device. The GPU used in this work clearly outperforms the computational power available today even in a high-end wearable system (e.g., a multicore smartphone). However, DeepConvLSTM achieves a recognition speed of 900× real-time using 1664 GPU cores at 1050 MHz. High-end mobile platforms already contain GPUs that can be used for general purpose processing [47]. A mobile processor, such as the Qualcomm Snapdragon 820, comprises 256 GPU cores running at 650 MHz and supports OpenCL profiles for general purpose GPU computing. While cores differ in capabilities, the available computational power may well be sufficient for real-time recognition in upcoming mobile devices. Training, however, is best envisioned server-side (e.g., as in [46]).

Removing the dependency on engineered features by exploiting convolutional layers is particularly important if the set of activities to recognise is changing over time, for instance as additional labelled data become available (e.g., through crowd-sourcing [48]). In such an “open-ended” learning scenario, where the number of classes could be increased after the system is initially deployed, backpropagation of the gradient to the convolutional layer could be used to incrementally adapt the kernels according to the new data at runtime. Future work may consider the representational limits of such networks for open-ended learning and investigate rules to increase network size (e.g., adding new kernels) to maintain a desired representational power.

## 6. Conclusions

In this paper, we demonstrated the advantages of a deep architecture based on the combination of convolutional and LSTM recurrent layers to perform activity recognition from wearable sensors. This new framework outperformed previous results in the OPPORTUNITY dataset of everyday activities by 4% on average and by 9% in an 18-class gesture recognition task. For the Skoda car manufacturing activity dataset, it outperformed previous deep non-recurrent approaches, improving the best reported scores by 6%. This extends the known domain of applicability for this unified framework of convolutional and recurrent units, which has never been reported on wearable sensor data.

In terms of time requirements, the recurrent architecture offers a very good trade-off between performance and training/recognition time when compared to a standard CNN. Indeed, the increase in training and recognition time for the recurrent architecture is only 20%.

We demonstrated that the recurrent LSTM cells are fundamental to allow one to distinguish gestures of a similar kind (e.g., “Open/Close Door 2” or “Open/Close Drawer 3”), which differ only by the ordering of the sensor samples. The baseline CNN model in comparison provided much worse performance on activities such as “Open/Close Door 2” or “Open/Close Drawer 3”, where it made 2–3-times more errors. Convolution kernels are only able to capture temporal dynamics within the duration of the kernel. In comparison, the recurrent LSTM cells do not have this limitation and can learn the temporal dynamics on various (potentially much longer) time scales depending on their learned parameters. Furthermore, when directly compared, we have showed how a recurrent architecture offers better segmentation characteristics than a standard CNN, being capable of defining the boundaries of activities much more precisely. These findings support the hypothesis that the LSTM-based model takes advantage of learning the temporal feature activation dynamics, which CNNs are not fully capable of modelling.

From the results, we can also see how the model is able to learn from signals obtained from accelerometers, gyroscopes and magnetometers, fusing them without requiring any specific preprocessing. The model offers a 0.69 $F_{1}$ score performance using only accelerometers. This performance improves on average by 15% when fusing accelerometers and gyroscopes and by 20% when fusing accelerometers, gyroscopes and magnetic sensors. This offers a trade-off for applications where sensor data from different sources must be automatically processed.

As future work, we will investigate a transfer learning approach based on these models to perform activity recognition on large-scale data. We propose to reuse kernels trained on the benchmark datasets for feature extraction. This potential transfer of features will ease the deployment of activity recognizers on cloud infrastructures.

The code and model parameters of DeepConvLSTM are available at [49].
